# Relation between mean arterial pressure and renal function in the early phase of shock: a prospective, explorative cohort study

**DOI:** 10.1186/cc10253

**Published:** 2011-06-06

**Authors:** Julie Badin, Thierry Boulain, Stephan Ehrmann, Marie Skarzynski, Anne Bretagnol, Jennifer Buret, Dalila Benzekri-Lefevre, Emmanuelle Mercier, Isabelle Runge, Denis Garot, Armelle Mathonnet, Pierre-François Dequin, Dominique Perrotin

**Affiliations:** 1 Service de Réanimation Médicale, Hôpital La Source, Centre Hospitalier Régional, avenue de l'Hôpital, 45067 Orléans Cedex 1, France; 2 Service de Réanimation Polyvalente - Hôpital Bretonneau-CHRU, 2 Boulevard Tonnellé, Tours, 37044, France

## Abstract

**Introduction:**

Because of disturbed renal autoregulation, patients experiencing hypotension-induced renal insult might need higher levels of mean arterial pressure (MAP) than the 65 mmHg recommended level in order to avoid the progression of acute kidney insufficiency (AKI).

**Methods:**

In 217 patients with sustained hypotension, enrolled and followed prospectively, we compared the evolution of the mean arterial pressure (MAP) during the first 24 hours between patients who will show AKI 72 hours after inclusion (AKI_h72_) and patients who will not. AKI_h72 _was defined as the need of renal replacement therapy or "Injury" or "Failure" classes of the 5-stage RIFLE classification (Risk, Injury, Failure, Loss of kidney function, End-stage renal disease) for acute kidney insufficiency using the creatinine and urine output criteria. This comparison was performed in four different subgroups of patients according to the presence or not of AKI at the sixth hour after inclusion (AKI_h6 _as defined as a serum creatinine level above 1.5 times baseline value within the first six hours) *and *the presence or not of septic shock at inclusion.The ability of MAP averaged over H6 to H24 to predict AKI_h72 _was assessed by the area under the receiver operating characteristic curve (AUC) and compared between groups.

**Results:**

The MAP averaged over H6 to H24 or over H12 to H24 was significantly lower in patients who showed AKI_h72 _than in those who did not, only in septic shock patients with AKI_h6_, whereas no link was found between MAP and AKI_h72 _in the three others subgroups of patients. In patients with septic shock plus AKI_h6_, MAP averaged over H6 to H24 or over H12 to H24 had an AUC of 0.83 (0.72 to 0.92) or 0.84 (0.72 to 0.92), respectively, to predict AKI_h72 _. In these patients, the best level of MAP to prevent AKI_h72 _was between 72 and 82 mmHg.

**Conclusions:**

MAP about 72 to 82 mmHg could be necessary to avoid acute kidney insufficiency in patients with septic shock and initial renal function impairment.

## Introduction

Acute circulatory failure is the main cause of renal failure in intensive care unit (ICU) patients [1-3], as low cardiac output and/or low mean arterial pressure (MAP) can cause low renal blood flow (RBF) and harm the kidney [4].

Very low levels of MAP are known to increase the risk of acute renal insufficiency (AKI) occurrence [5-7]. In counterpart, although a MAP of at least 65 mmHg is thought to be protective against organ failures, including renal impairment, and is universally recommended [8], the true value of MAP that could really protect renal function against worsening is still unknown. In human septic shock, two interventional prospective studies of limited size [9,10] have shown that increasing MAP from 65 to 75 or 85 mmHg with norepinephrine did not result in urinary output nor serum creatinine significant improvement. Conversely, in another recent study [11], increasing MAP from 65 to 75 mmHg resulted in an increase in urinary output in 11 septic shock patients. Further, a recent retrospective cohort study suggested that levels of MAP higher than 75 mmHg could be necessary to insure renal protection during sepsis and septic shock [12].

In healthy conditions RBF is stable within a wide range of MAP, due to regional autoregulation [13]. However, in shock states, and particularly in septic shock, derangements in microcirculation and vasoreactivity, although not precisely examined for human renal perfusion, tend to increase the lowest MAP threshold that guarantees autoregulation [14]. In addition, animal studies have shown that in ischemic AKI, a decrease in MAP even at high levels of MAP (above 90 mmHg) could be responsible of a decrease in RBF, suggesting an impairment of autoregulation in AKI [15-18]. This suggests that independently of the shock state itself, shock-induced AKI could cause the partial or total loss of the renal autoregulation ability early in the course of the disease [17]. This could explain the discrepancies between the above-cited human studies [9-12] concerning the relation between MAP and renal function as they included patients regardless of the existence or not of AKI at the time of inclusion. However, at this time, human studies addressing the link between shock-induced AKI and the loss of autoregulation are lacking.

We hypothesized that, due to different MAP thresholds of renal autoregulation, patients with or without AKI at the time of initial therapy for acute circulatory failure could need different MAP levels to prevent the worsening of renal function or even to favour its improvement.

Accordingly, we conducted an explorative, prospective study aimed at comparing the relation between MAP and renal function in patients admitted for acute circulatory failure with different degrees of initial renal function impairment.

## Materials and methods

The protocol met the criteria of a noninterventional study design as defined by the French Law [19]. The Ethics Committee of the *Association des Réanimateurs du Centre-Ouest, France *approved the protocol and waived informed consent. The study was conducted in two intensive care units (ICUs) (one medical ICU in a university hospital, one medical-surgical ICU in a regional hospital) between October 2007 and April 2009. In both ICUs treatment of acute circulatory failure followed national and international guidelines, especially concerning septic shock. For all shock states, first line therapies were prompt vascular volume expansion in case of probable hypovolemia, immediate antibiotics in case of sepsis, invasive mechanical ventilation if necessary, quick use of continuous iv norepinephrine to reach a MAP level above 65 mmHg, systematic echocardiography within the first hours, systematic dobutamine use in case of systolic myocardial dysfunction or low superior vena cava oxygen saturation after volume expansion.

Patients with hypotension (defined as a systolic arterial pressure below 90 mmHg and/or a MAP below 65 mmHg over 10 minutes) for less than 12 h were included at the time (H1) they were carrying an arterial line and a bladder catheter. Patients were not included in case of renal transplant, chronic haemodialysis, diabetic ketoacidosis, or diabetes insipidus. Patients were excluded if they died or were discharged before the ninth hour after inclusion (H9), if they were started on renal replacement therapy (RRT) before H9, or on diuretics before H9, if the arterial and/or bladder catheters were removed before H12, or if diabetes insipidus occurred between H1 and H72.

### Data collection

We recorded age, gender, size, body weight, underlying diseases (chronic hypertension, diabetes mellitus, chronic cardiac failure, liver cirrhosis, chronic renal insufficiency (defined as steady state creatinine clearance < 60 mL/minute), presence of solitary kidney), use of antihypertensive drugs before admission, type of antihypertensive drug used (angiotensin conversion enzyme (ACE) inhibitors; angiotensin II receptor blockers (ARB); diuretics, calcium inhibitors), administration of nonsteroidal anti-inflammatory drugs (NSAID), immunoglobulins, methotrexate, lithium, aciclovir, amphotericin, ciclosporin, tacrolimus, cisplatin, or protease inhibitors within 72 hours before inclusion, aminoglycosides or vancomycin within 96 hours before inclusion, iodinated contrast media within five days before inclusion, number of nephrotoxic drugs received before inclusion, presence of an urinary tract obstruction or not, urinary origin of sepsis, cause of shock (septic [20], cardiogenic, haemorrhagic, hypovolemic), simplified acute physiology score (SAPS II) [21], ICU and hospital stay outcome. Recent serum creatinine at steady state was searched for all patients in hospital electronic registry and by calling the generalist practitioner. In case of an unsuccessful search, steady state serum creatinine was determined by the MDRD formula [22].

We also recorded the time elapsed between the beginning of hypotension and inclusion, the lowest MAP recorded before inclusion and the volume of vascular expansion within the six hours before inclusion.

MAP and urine output, and catecholamine dosages were recorded hourly from H1 to H72. Serum creatinine was measured at least once between H1 and H6, at H12 and then every 12 hours during the observation period and at ICU discharge.

All the above variables were collected prospectively. Training courses were performed in the two centres, for physicians and ICU nurses, before the beginning of the study, with particular attention paid to the recording of time events (first hypotension, inclusion), to the recording of hourly MAP and urine output, and to the timing of blood sampling (for serum creatinine measurement at H1, H6 and then every 12 hours until H72 or ICU discharge).

### Definitions and study endpoint

At H6 the patients were classified in two predefined groups according to the creatinine criterion of the RIFLE classification [22] taking into account the highest value of serum creatinine between H1 and H6: 1) patients of the "noAKI" class (noAKI_h6 _patients); 2) patients of the "Risk", "Injury" or "Failure" classes (AKI _h6 _patients).

The study endpoint was the presence or not of AKI at H72. At this time patients were considered as suffering from AKI if they were in the classes "Injury" or "Failure" (or have been started on renal replacement therapy) (AKI _h72 _patients), based on the RIFLE classification including the creatinine and the urine output criteria. We considered patients classified as "noAKI" or "Risk" at H72 as not suffering from AKI at this time (noAKI _h72 _patients).

### Data analysis

In each patient group (noAKI_h6 _patients and AKI_h6 _patients), we compared hourly MAP at each time-point from H6 to H24 between patients who showed AKI at H72 and those who did not, by two-factor analysis of variance (ANOVA) for repeated measurements. In case of a significant link between AKI at H72 and sequential MAP values as disclosed by ANOVA, a *post hoc *t-test was used to find out time-points at which MAP was significantly different between noAKI_h72 _and AKI_h72 _patients.

In each patients group (noAKI_h6 _patients and AKI_h6 _patients) we examined the ability of MAP averaged over H6 to H24 and over H12 to H24 to predict AKI at H72 by calculating the area under the receiver operating characteristic curve (AUC), determining the best threshold (Youden's method) [23], sensitivity, and specificity. In addition to the best threshold we provide the highest MAP threshold yielding a positive likelihood ratio (LR) > 5 and the lowest MAP threshold yielding a negative LR < 0.2. AUCs were compared between groups [24]. As septic shock alone represents a well-identified cause of AKI [3], we also examined the value of MAP to predict AKI at H72 in the specific group of patients with septic shock. Finally, we also searched for different relationships between MAP and AKI at H72 according to the presence or not of chronic hypertension.

AUCs, sensitivity and specificity are given with their 95% confidence intervals (95CI). Continuous variables are expressed as mean ± SD unless otherwise specified. A value of *P *< 0.05 was considered significant. All statistical tests were two-tailed, performed using MedCalc^® ^(Mariakerke, Belgium) and Statview^® ^(SAS Institute, Cary, NC, USA).

## Results

**Figure 1:**
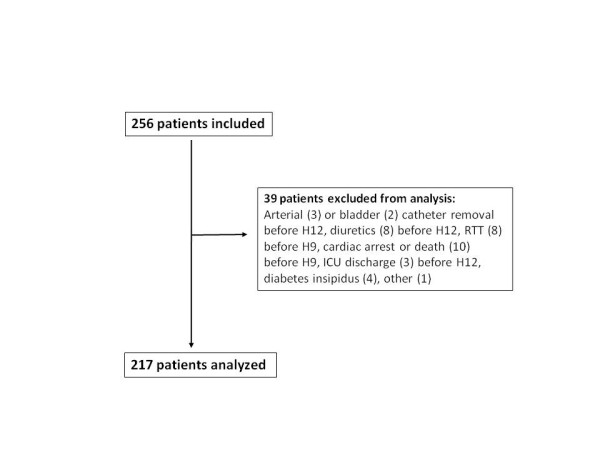
**Flow diagram**. RRT: renal replacement therapy.

Among the 256 patients enrolled, 39 met at least one exclusion criteria (Figure 1) and 217 were analysed (Table 1). The duration of the hourly urine output and MAP collection period was 49 ± 19 hours. Eighteen patients (8.3%) were started on RRT before H72 and no other patient required RRT between H72 and ICU discharge.

**Table 1:** Characteristics of the 217 patients analysed

	Entire population N = 217	Patients without AKI at H6 N = 116	Patients with AKI at H6 N = 101
**Age (years)**	64 ± 15	64 ± 16	64 ± 15
**Sex Male, n (%)**	127 (59%)	76 (66%)	51 (50%)*
**SAPSII on admission**	53.2 ± 18	50.2 ± 16	56.7 ± 16*
**Weight (kg)**	73 ± 18	75 ± 20	71 ± 16
**Size (cm)**	165 ± 15	165 ± 15	165 ± 15
**Underlying diseases:**			
hypertension, n (%)	90 (41%)	48 (41%)	42(41%)
type 1 diabetes, n (%)	2 (1.0%)	2 (2%)	0 (0%)
type 2 diabetes, n (%)	33 (15%)	15 (13%)	18 (18%)
chronic cardiac failure, n (%)	27 (12.0%)	12 (10%)	15 (15%)
liver cirrhosis, n (%)	9 (4%)	5 (4%)	4 (4%)
past history of acute renal failure, n (%)	10 (5%)	6 (5%)	4 (4%)
chronic renal failure n (%)	9 (4%)	3 (3%)	6 (6%)
**Antihypertensive drugs regularly taken**			
ACE inhibitors, n (%)	41 (19%)	22 (20%)	19 (19%)
ARBs, n (%)	22 (10%)	12 (10%)	10 (10%)
Calcium- channels blockers, n (%)	10 (5%)	5 (4%)	5 (5%)
Diuretics, n (%)	55 (25%)	29 (25%)	26 (26%)
**Nephrotoxic drugs**			
NSAID within 72 hours before inclusion, n (%)	9 (4%)	3 (3%)	6 (6%)
Aminoglycosids in the last 96 h, n (%)	52 (24%)	26 (22%)	26 (26%)
Vancomycin in the last 96 h, n (%)	14 (6%)	4 (3%)	10 (10%)*
Iodine containing contrast media in the last five days, n (%)	35 (16%)	20 (17%)	15 (15%)
**Cause of acute circulatory failure:**			
Septic shock, n (%)	127 (59%)	64 (55%)	63 (62%)
Cardiogenic shock, n (%)	18 (8%)	10 (9%)	8 (8%)
Hemorrhagic shock, n (%)	9 (4%)	4 (3%)	5 (5%)
Hypovolemic shock, n (%)	42 (20%)	27 (23%)	17 (17%)
Post cardiac arrest (%)	15 (6%)	9 (8%)	6 (6%)
Unknown n (%)	5 (2%)	3 (3%)	2 (2%)
**Urinary sepsis, n (%)**	26 (12%)	10 (9%)	16 (16%)
**Urinary tract obstruction, n (%)**	8 (4%)	3 (3%)	2 (2%)
**Acute kidney insufficiency at H72, n (%)**	66 (30%)	23 (20%)	43 (43%)*
**ICU death, n (%)**	76 (35%)	40 (34%)	36 (36%)
**Hospital death, n (%)**	84 (39%)	42 (36%)	42 (42%)
**Time elapsed between occurrence of hypotension and inclusion (hours)**	4.1 ± 4.4 (median = 3; IQR: 1.5 to 6.5)	4.0 ± 4.5	4.3 ± 4
**Time elapsed between occurrence of hypotension and inclusion (hours)**	4.1 ± 4.4 (median = 3; IQR: 1.5 to 6.5)	4.0 ± 4.5	4.3 ± 4
**Lowest MAP before inclusion (mmHg)**	52 ± 13 mm Hg (median = 53; IQR: 44 to 62)	52 ± 14	52 ± 14
**MAP at inclusion (mmHg)**	68 ± 16 (median = 66; IQR: 57 to 76)	67 ± 15	68 ± 17
**Continuous i.v. catecholamines during the first 72 hours**			
**Epinephrine, n (%)**	2 (1%)	0 (0%)	2 (2%)
**Norepinephrine alone, n (%)**	107 (43.5%)	54 (43.5%)	53 (43.5%)
**Dobutamine alone, n (%)**	1 (4%)	0 (4%)	1 (4%)
**Dobutamine + Norepinephrine, n (%)**	10 (5%)	8 (5%)	2 (5%)
**Epinephrine+ Norepinephrine, n (%)**	7 (2%)	2 (2%)	5 (2%)
**Epinephrine + Norepinephrine + Dobutamine, n (%)**	2 (17%)	1 (17%)	1 (17%)
**None, n (%)**	88 (28)	51 (28)	37 (28)
**Volume expansion in the last six hours before inclusion (mL)**	2,190 ± 1,690 (median = 2,000; IQR: 1,000 to 3,000)	1,960 ± 1,720	2,460 ± 1,600*
**Volume expansion from six hours before inclusion** **to H72 (mL)**	4,800 ± 2,660 (median = 4,500; IQR: 3,000 to 6,000)	4,450 ± 2,730	5,180 ± 2,540

ACE: Angiotensin Conversion Enzyme; AKI: Acute Kidney Insufficiency; ARB: Angiotensin II receptor blockers; IQR: interquartile range; MAP: mean arterial pressure; NSAID: Non Steroidal Anti Inflammatory Drug.

*: Significant difference between patients with AKI at H6 and patients without AKI at H6 (*P *< 0.05)

At H6, 116 patients were classified in the noAKI _h6 _group and 101 patients in the AKI _h6 _group. A total of 66 patients showed AKI at H72: 23 among the noAKI_h6 _patients (20%) and 43 among the AKI_h6 _patients (43%) (*P *= 0.0004). Table 2 shows the repartition of the patients among the different RIFLE classes at H6 and at H72.

**Table 2:** Repartition of the 217 patients among the different RIFLE classes at H6 and then at H72

RIFLE class at H6 *based on creatinine criterion only (highest serum creatinine between H1 and H6)*	RIFLE class at H72 *based on creatinine and urine output criteria*			
	**noAKI**	**Risk**	**Injury**	**Failure or RRT**

**noAKI**, n = 116	86 (74%)	7 (6%)	14 (12%)	9 (8%)
**Risk**, n = 38	18 (47%)	9 (24%)	7 (18%)	4 (11%)
**Injury**, n = 39	15 (38%)	5 (13%)	8 (21%)	11 (28%)
**Failure**, n = 24	9 (38%)	2 (8%)	3 (13%)	10 (42%)

AKI: Acute kidney insufficiency; RIFLE refers to of the 5-stage RIFLE classification (Risk, Injury, Failure, Loss of kidney function, End-stage renal disease) for acute kidney insufficiency; RRT: Renal replacement therapy.

In 62 patients out of 217 (29%) the baseline serum creatinine could not be retrieved and was estimated by the MDRD formula. These patients were not different from the patients with known baseline serum creatinine with regard to the percentage of AKI at H6 (23 (37%) vs 78 (50%), respectively; *P *= 0.1) or of AKI at H72 (16 (26%) vs 50 (32%), respectively; *P *= 0.4).

### Comparison of MAP between patients groups

The two-factor ANOVA for repeated measurements examining MAP from H6 to H24 showed that MAP was significantly different between noAKI_h72 _patients and AKI_h72 _patients (*P *= 0.016) and between noAKI_h6 _patients and AKI_h6 _patients (*P *= 0.043). The analysis disclosed the same results when re-run with MAP from H12 to H24 as the dependent variable, and in addition showed a significant interaction between the factors AKI at H6 and AKI at H72 (*P *= 0.049). When re-run in the sub-group of patients with AKI at H6, with AKI at H72 and septic shock as the two independent variables, this analysis showed that MAP from H6 to H24 was significantly different between patients with and without AKI at H72 (*P *= 0.01) and that AKI at H72 and septic shock interacted to influence MAP (*P *= 0.02). These results allowed us to compared MAP at each time point between the different sub-groups of patients.

**Figure 2:**
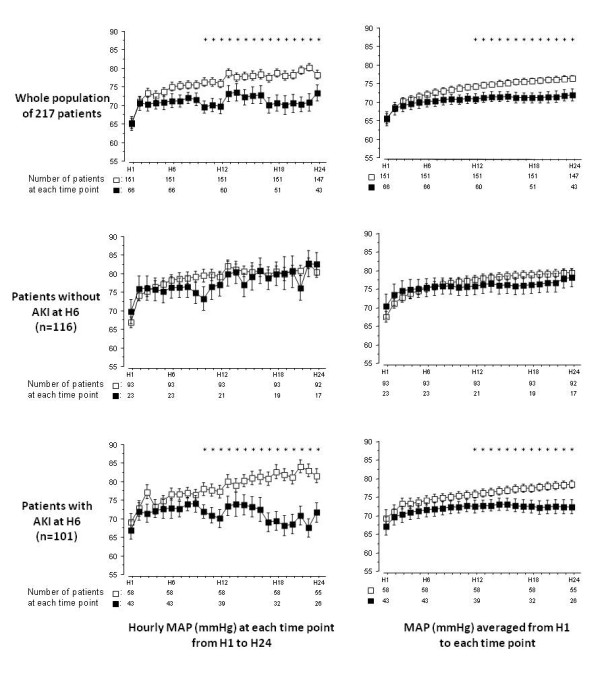
**Evolution of mean arterial pressure (MAP) during the first 24 hours**. The evolution of hourly MAP (left panels) and of MAP time-averaged MAP (right panels) compared between patients who will have acute kidney insufficiency (AKI) at H72 (black squares) and those who will not (open squares), is shown for the whole population (top panels), for the group of patients with no AKI at H6 (middle panels) and for the group of patients with AKI at H6 (bottom panels). The significant differences observed in MAP (from H10 to H24 for hourly MAP and from H12 to H24 for time-averaged MAP, as indicated by an asterisk upon each time point) between patients who will or will not have AKI at H72 in the whole population (top panels) were mainly due to the patients with AKI at H6 (bottom panels). Asterisks upon time points indicate a significant difference (*P *< 0.05) between patients who will have AKI at H72 (black squares) and those who will not (open squares) (*post hoc *comparison after analysis of variance). Error bars represent standard errors.

**Figure 3:**
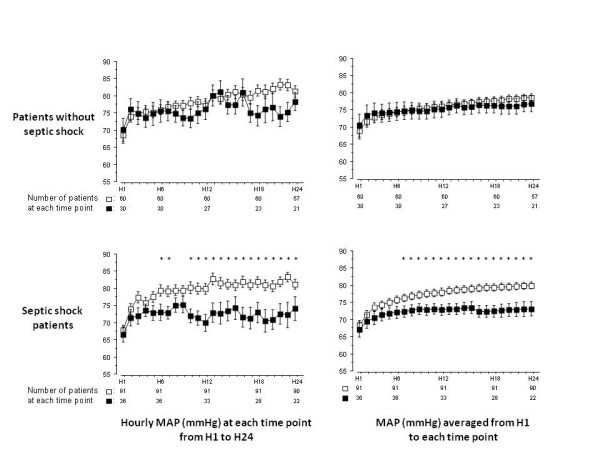
**Mean arterial pressure (MAP) according to the presence or not of septic shock**. The MAP (from H6 to H24 for hourly MAP and for time-averaged MAP) was significantly lower in patients who will than in those who will not have AKI at H72 in the septic shock population (as indicated by an asterisk upon each time point) (bottom panels), while no difference was found in the non septic shock patients (top panels). Asterisks upon time points indicate a significant difference (*P *< 0.05) between patients who will have AKI at H72 (black squares) and those who will not (open squares) (*post hoc *comparison after analysis of variance). Error bars represent standard errors.

**Figure 4:**
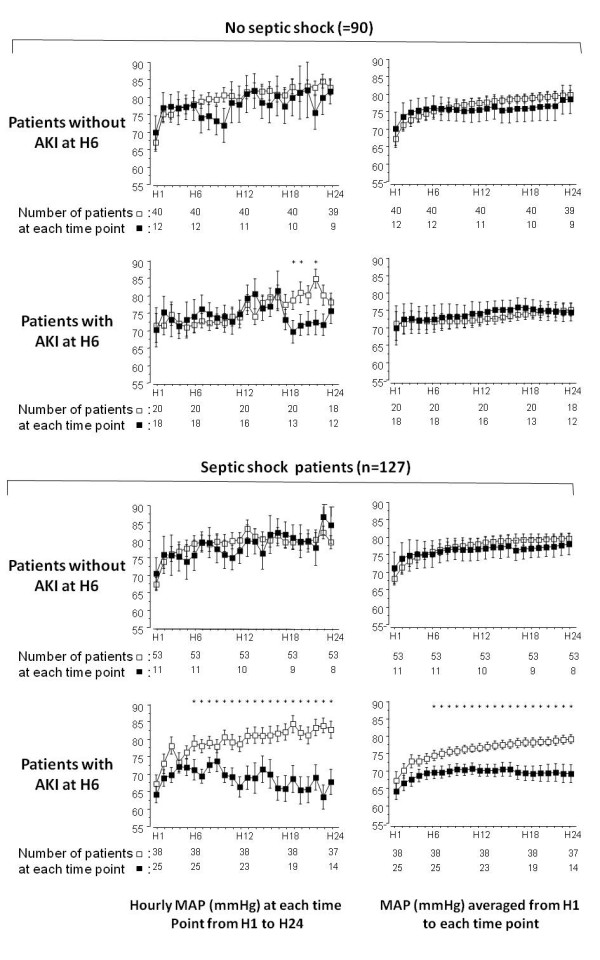
**Mean arterial pressure (MAP) according to the presence of septic shock or acute kidney insufficiency**. The MAP (from H6 to H24 for hourly MAP and for time-averaged MAP) was significantly lower in patients who will than in those who will not have AKI at H72 *only *in the sub-group of patients with septic shock and AKI at H6 (as indicated by an asterisk upon each time point in the bottom panels). Asterisks upon time points indicate a significant difference (*P *< 0.05) between patients who will have AKI at H72 (black squares) and those who will not (open squares) (*post hoc *comparison after analysis of variance). Error bars represent standard errors.

As illustrated in Figure 2, in the AKI_h6 _patients, MAP at each time point between H10 and H24 was significantly lower in patients who had AKI at H72 than in those who did not. In the noAKI_h6 _patients, MAP did not differ between patients who had AKI at H72 than in those who did not. As shown in Figure 3 this difference in MAP evolution between noAKI_h72 _patients and AKI_h72 _patients was also retrieved in the population of septic shock patients (n = 127) but not in patients with non septic shock. As illustrated in Figure 4, when MAP evolution was compared between noAKI_h72 _patients and AKI_h72 _patients according to the presence or not of AKI at H6 and of septic shock, it appeared that MAP from H6 to H24 was lower in the patients who will show AKI at H72 than in patients who will not, *only *in the subgroup of patients with septic shock *and *AKI at H6.

**Figure 5:**
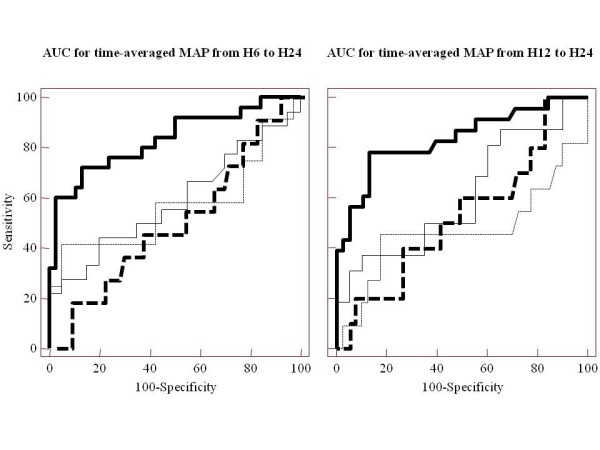
**Performance of mean arterial pressure to predict acute kidney insufficiency (AKI) at H72**. The areas under the receiver operating characteristics curves (AUC) of time-averaged MAP over H6 to H24 (left panel) and over H12 to H24 (right panel) to predict acute kidney insufficiency (AKI) at H72 was examined in four subgroups of patients: patients with no AKI at H6 and non septic shock (black thin line), patients with no AKI at H6 and septic shock (dashed thick line), patients with AKI at H6 and non septic shock (dashed thin line), and patients *with AKI at H6 and septic shock *(black thick line). In this latter subgroup, the AUC (see values in Table 3) was significantly higher than in the three others subgroups for time-averaged MAP over H6 to H24 (left panel) (*P *= 0.0037 *vs *the no AKI at H6 and septic shock patients; *P *= 0.0037 *vs *the no AKI at H6 and non septic shock patients; *P *= 0.02 *vs *the AKI at H6 and non septic shock patients) and over H12 to H24 (right panel) ((*P *= 0.0065 *vs *the no AKI at H6 and septic shock patients; *P *= 0.002 *vs *the no AKI at H6 and non septic shock patients; *P *= 0.036 *vs *the AKI at H6 and non septic shock patients). MAP: mean arterial pressure.

Table 3 shows the AUCs for time-averaged MAP to predict AKI at H72 in the different groups of patients. It appears that time-averaged MAP predicted AKI at H72 with good discriminative power only in the sub-group of patients with AKI at H6 and septic shock. In this sub-group time-averaged MAP over H6 to H24 yielded an AUC of 0.83 (0.72 to 0.92) associated with a sensitivity of 0.72 and specificity of 0.87. The best cut-off value of time-averaged MAP over H6 to H24 was 72 mmHg, which was also the highest value associated with a positive LR > 5 (5.5 (4.2 to 7.2)). The lowest value of time-averaged MAP over H6 to H24 associated with a negative LR < 0.2 was 80 mmHg (negative LR = 0.16 (0.04 to 0.6), sensitivity = 0.92 (0.74 to 0.99), specificity = 0.50 (0.33 to 0.67)). In the same sub-group of patients time-averaged MAP over H12 to H24 yielded an AUC of 0.84 (0.72 to 0.92) associated with a sensitivity of 0.78 and specificity of 0.89. The best cut-off value of time-averaged MAP over H12 to H24 was 72 mmHg, which was also the highest value associated with a positive LR > 5 (6.0 (4.6 to 7.6)). The lowest value of time-averaged MAP over H12 to H24 associated with a negative LR < 0.2 was 82 mmHg (negative LR = 0.19 (0.05 to 0.8), sensitivity = 0.92 (0.74 to 0.99), specificity = 0.50 (0.33 to 0.67)). Figure 5 shows the different AUCs of time-averaged MAP over H6 to H24 or over H12 to H24 to predict AKI at H72 in the four sub-groups of patients according to the presence or not of AKI at H6 and of septic shock.

**Table 3:** Discriminative power of mean arterial pressure to predict acute kidney insufficiency at H72

	AUC for MAP averaged over H6 to H24	Best MAP threshold (mmHg)	Sensitivity	Specificity	AUC for MAP averaged over H12 to H24	Best MAP threshold (mmHg)	Sensitivity	Specificity
Patients with no AKI at H6 (n = 116)	0.53 (0.43 to 0.62)	-	-	-	0.50 (0.41 to 0.60)	-	-	-
Patients with AKI at H6 (n = 101)	0.74* (0.66 to 0.83)	69	0.53 (0.38 to 0.69)	0.91 (0.81 to 0.97)	0.75** (0.66 to 0.84)	70	0.59 (0.42 to 0.74)	0.86 (0.75 to 0.94)
Patients with non septic shock (n = 90)	0.60 (0.49 to 0.11)	-	-	-	0.59 (0.48 to 0.59)	-	-	-
Septic shock patients (n = 127)	0.72 (0.63 to 0.79)	72	0.56 (0.38 to 0.72)	0.84 (0.74 to 0.91)	0.72 (0.63 to 0.80)	72	0.61 (0.42 to 0.77)	0.84 (0.74 to 0.91)
***Non septic shock patients only:***								
Patients with no AKI at H6 (n = 52)	0.57 (0.42 to 0.71)	-	-	-	0.47 (0.33 to 0.61)	-	-	-
Patients with AKI at H6 (n = 38)	0.59 (0.42 to 0.74)	-	-	-	0.61 (0.44 to 0.77)	-	-	-
***Septic shock patients only:***								
Patients with no AKI at H6 (n = 63)	0.52 (0.39 to 0.64)	-	-	-	0.53 (0.40 to 0.66)	-	-	-
Patients with AKI at H6 (n = 64)	0.83^†,††^ (0.72 to 0.92)	72	0.72 (0.51 to 0.88)	0.87 (0.72 to 0.96)	0.84^‡,‡‡^ (0.72 to 0.92)	72	0.78 (0.56 to 0.93)	0.89 (0.72-0.96)

*: *P *= 0.011 versus patients with no AKI at H6; **: *P *= 0.003 versus patients with no AKI at H6; **^†^**: *P *= 0.0037 versus septic shock patients with no AKI at H6;

^††^: *P *= 0.02 versus non septic shock patients with AKI at H6; **^‡^**: *P *= 0.0065 versus septic shock patients with no AKI at H6; ^‡‡^: *P *= 0.036 versus non septic shock patients with AKI at H6

AKI: Acute kidney insufficiency; AUC: Area under the receiver operating characteristics curve; MAP: Mean arterial pressure.

**Figure 6:**
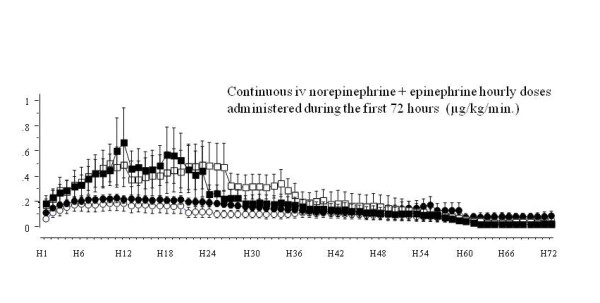
**Vasopressors doses administered during the first 72 hours**. To draw this figure we summed the hourly doses of norepinephrine and epinephrine (μg/kg/min.) administered continuously by iv infusion, considering these two catecholamines as equipotent in term of vasopressor activity. It shows that the doses of vasopressor administered were higher in patients who will show acute kidney insufficiency at H72 (squares) compared to those who will not (circles), particularly during the first 24 hours, and that this difference was retrieved in septic shock (black squares and circles) and in non septic shock patients (open squares and circles).

The patients who had AKI at H72 received higher doses of vasopressors (continuous iv epinephrine and/or norepinephrine) during the first 72 hours, a difference especially marked during the first 24 hours as illustrated in Figure 6. However, during the first 72 hours, 158 patients did not receive a dosage of vasopressors above 0.5 μg/kg/minute during at least two hours. Among these patients we considered as not receiving high doses of vasopressors, 41 patients had AKI at H72 and among them, 39% (16/41) had a MAP averaged over H12 to H24 below 72 mmHg.

## Discussion

The main result of this prospective study is that in shocked patients with initial renal insult as defined by a serum creatinine above 1.5 times the baseline value during the first 6 hours, the occurrence of AKI during the first 72 hours of care is linked to the time-averaged MAP obtained with therapy during the first 24 hours. In these patients, it appears that a MAP higher than the universally recommended level of 65 mmHg could be necessary to avoid worsening of renal function. Our subgroup analyses revealed that this is especially true in septic shock patients, whereas in patients with shock of other origins the link between MAP and AKI at H72 is less obvious.

MAP level is essential to protect renal function, since below a certain MAP threshold, when autoregulation is exceeded, RBF decreases and leads to AKI [25]. In our study, the best threshold of time-averaged MAP over H6 to H24 or over H12 to H24 for predicting AKI at H72, ranged from 72 to 82 mmHg in patients with AKI at H6 and septic shock. This result is in line with the retrospective study conducted by Dunser *et al*. [12], which proposed that the best MAP threshold for predicting the need of RRT in septic shock was 75 mm Hg. Altogether, these observations suggest that patients with septic shock and initial renal insult might need a higher MAP than patients with lower risk of AKI. This could be explained by the loss of autoregulation following acute renal insult. Nevertheless, up to now clinical data to support this view are still lacking. Only animal studies about ischemic acute renal failure induced by the clamping of renal artery have shown an impairment of autoregulation from the 18th hour following the renal injury [15-18].

In our study, in the patients with non septic shock and/or no AKI at H6, we did not determine any MAP threshold that could predict AKI at H72. Perhaps in these patients autoregulation was preserved. Furthermore, MAP is not the sole determinant of renal function in shock. Organ hypoperfusion and/or reperfusion lead to an important inflammatory reaction and inflammatory mediator systemic delivery known to be involved in renal injuries [26-30]. The recent study conducted by Lerolle *et al*. [31] confirms that renal lesions associated with AKI in septic shock are more complex than acute tubular injuries, involving intense capillary leukocytic infiltration and apoptosis. Moreover, aetiology of AKI in ICU patients is often multifactorial. The usual AKI risk factors identified are hypertension, diabetes, heart failure, sepsis [3,32] and a high score of illness severity [33], all of which being not amenable to medical intervention in the acute situation of shock. Given these considerations, increasing MAP could be insufficient to avoid AKI. At this time, waiting for further studies, our results suggest that in patients with septic shock and initial renal insult (and perhaps also in patients with initial renal insult without septic shock), higher levels than the universally recommended level of 65 mmHg could be targeted. This could be achieved with an increase in norepinephrine dosage, as it has not been shown to adversely affect renal perfusion [7,9,10,34,35]. Indeed the patients with low MAP are often those who also receive the highest doses of vasopressors, and consequently vasopressor dosages are statistically linked to AKI occurrence (as illustrated in Figure 6 for our patients). In consequence, for fear of precipitating AKI, one might be rather timid in increasing doses of vasopressors once the targeted MAP of 65 mmHg is attained. However, our analysis showed that a significant number of patients with rather low MAP (< 72 mmHg) averaged over H12 to H24 showed AKI at H72 while not receiving vasopressors at doses extraordinarily high (less than 0.5 μg/kg/min of epinephrine and/or norepinephrine). In our opinion, there was still room for an increase in vasopressor doses in these patients.

Current recommendations for the prevention of AKI in the ICU [36] propose to achieve a MAP above 60 to 65 mmHg but indicate that this target pressure should be individualized when possible, especially if knowledge of the premorbid blood pressure is available. In case of chronic hypertension, the autoregulation MAP thresholds are known to be higher than in non hypertensive patients, and this could suggest that higher levels of blood pressure are necessary in hypertensive patients to maintain RBF [37]. However, definitive clinical studies supporting this view are difficult to retrieve in the literature. In our study population, chronic hypertension was not a predisposing factor for AKI at H72, and we could not identify chronic hypertension as a condition needing higher MAP levels to avoid AKI (See Additional data file 1). A good control of hypertension by therapeutics before admission to the ICU could be one factor among others explaining this apparent preservation of autoregulation in hypertensive patients.

Our study has several limitations. First, it is an observational study in which the MAP level was not a controlled variable, and as a certain degree of colinearity unquestionably exists between the severity of disease and the MAP level, this colinearity was difficult to circumvent by our statistical analysis. However, prospective studies like ours are scarce in this field and our results add one more element to support the view that increasing MAP above 65 mmHg might be necessary at least in septic shock to prevent AKI. Second, we did not measure cardiac output, which is an important predictor of RBF [30], particularly in hypodynamic shock. However, its role during hyperdynamic shock is less crucial [38,39] and our population comprised a majority of hyperdynamic shocks like resuscitated septic shocks.

MAP is an important factor participating to AKI in shock, and probably its level should be adjusted for each individual patient, as suggested by our results and by others studies [11,12]. Nevertheless, improvement of macrocirculation may be insufficient to avoid shock-induced AKI as disturbances of renal microcirculation may persist even after restoration of optimal perfusion pressure and cardiac output [40-42]. Evaluation of renal perfusion with Doppler ultrasonography could help clinicians to improve hemodynamic management according to renal resistive index [11,43,44].

## Conclusions

We found that a threshold of MAP within 72 to 82 mmHg could be necessary to avoid AKI in septic shock with initial renal insult. Future randomized clinical trials are necessary to determine the MAP level to reach in shock (septic or not). Based on our observations, concerning the preservation of renal function, these trials should focus on patients with initial renal insult.

## Key messages

• In septic shock patients with initial renal insult, a time-averaged mean arterial pressure between 72 and 82 mmHg during the first 24 hours was associated with lower incidence of acute kidney insufficiency at H72.

• In septic shock patients with initial renal insult, a mean arterial pressure higher than the universally recommended level of 65 mmHg might reduce the risk of progression or persistence of acute kidney insufficiency.

## Abbreviations

ACE: angiotensin conversion enzyme inhibitors; AKI: acute renal insufficiency; AKI_h6_: acute kidney insufficiency at H6; AKI_h72_: acute kidney insufficiency at H72; ANOVA: analysis of variance; ARB: angiotensin II receptor blockers; AUC: area under the receiver operating characteristic curve; LR: likelihood ratio; MAP: mean arterial pressure; MDRD: Modification of the Diet in Renal Disease; NSAID: nonsteroidal anti-inflammatory drugs; RBF: renal blood flow; RIFLE classification: the Risk, Injury, Failure, Loss, and End-stage Kidney classification; RRT: renal replacement therapy; SAPS: simplified acute physiology score.

## Competing interests

The authors declare that they have no competing interests.

## Authors' contributions

TB, JuB and SE designed the study. TB, JuB, SE, PFD and DP wrote the manuscript. All authors participated in the enrolment of patients and in the acquisition of data. All authors declare they have read and approved the final manuscript.

## Supplementary Material

Additional file 1 **The interrelationships between chronic arterial hypertension, mean arterial pressure during shock, and the occurrence of Acute Kidney Insufficiency**. 1) Relation between chronic hypertension and Acute Kidney Insufficiency; 2) Evolution of mean arterial pressure during the first 24 hours compared between patients with and without Acute Kidney Insufficiency at H72, in subgroups of patients according to the presence or not of chronic hypertension. Click here for file
