# Supplementary material for: Relation between mean arterial pressure and renal function in the early phase of shock: a prospective, explorative cohort study
Source: Crit Care. 2011 Jun 6;15(3):R135. doi: 10.1186/cc10253 (PMC3219004; doi:10.1186/cc10253)

**Additional data file 1**

**1) Relation between chronic hypertension and Acute Kidney Insufficiency**

To examine the potential link between chronic arterial hypertension and the occurrence of acute kidney insufficiency (AKI) at H72 in our study population, we performed a multivariate stepwise logistic regression with the presence of AKI at H72 as the dependent variable and the clinical and laboratory data recorded within the first 24 hours that were linked to AKI at H72 with p<0.15 by univariate analysis (t test or Fisher exact test when appropriate) as potential predictors. **Table A1** shows the variables entered in this logistic regression and shows that only three variables remained significantly linked to AKI at H72 after multivariate analysis: the SAPSII (p=0.0001), the MAP averaged over H12 to H24 (p=0.003), and the highest serum creatinine between H1 and H6 (p=0.037).

To ascertain that the stepwise procedure did not miss some effect due to chronic hypertension we re-run the analysis by forcing the variable “chronic hypertension”, and again this analysis did not retain “chronic hypertension” as a predictor of AKI at H72.

We then search for potential interactions between “chronic hypertension”, “septic shock”, and the “presence of AKI at H6”. All possible combinations of these three variables were entered as dummy variables in the multivariate regression analysis with stepwise design. **Table A2** shows that the variables “at least 2 conditions among chronic hypertension, septic shock, and presence of AKI at H6”, liver cirrhosis, and “Septic shock *and* AKI at H6” were retained by the analysis in addition to the variables SAPSII, MAP averaged over H12 to H24, and highest serum creatinine between H1 and H6.

It must be noted that in our last analysis (**Table A2**) the association between AKI at H6 and septic shock appeared to protect against AKI at H72, a surprising effect difficult to explain in view of the classical risk of AKI in septic shock patients. Our interpretation of this observation is that it was probably the result of an overanalysis of our data (too many variables entered in the model with regard to the limited size of the study population).

Finally, at the end of the analyses, it was concluded that in our study population the presence of chronic hypertension was not linked to AKI at H72.

**Table A1:** Risk factors for acute kidney insufficiency at H72

|  | No AKI***** at H72:  n=151 | AKI at H72*****:  n=66 | p value  for univariate comparison | Adjusted Odd ratios for variables remaining as potential predictors after multivariate logistic regression |
| --- | --- | --- | --- | --- |
| MAP averaged over H1 to H12 (mmHg) | 77 ± 10 | 73 ± 10 | 0.015 | - |
| MAP averaged over H1 to H24 (mmHg) | 79 ± 9 | 73 ± 11 | 0.0001 | - |
| MAP averaged over H6 to H24 (mmHg) | 80 ± 10 | 74 ± 12 | <0.0001 | - |
| MAP averaged over H12 to H24 (mmHg) | 81 ± 11 | 73 ± 15 | <0.0001 | 0.95 (0.92- 0.98)  (for 1 mmHg) |
| Use of NSAIDs | 4 (3%) | 5 (8%) | 0.13 | - |
| Highest serum creatinine between H1 and H6 (µmom/L) | 135 ± 90 | 197 ± 131 | <0.0001 | 1.004 (1.002-1.008)  (for 1 µmol/L) |
| Liver cirrhosis | 3 (2%) | 6 (9%) | 0.05 | - |
| More than 0.5 µg/kg/min of epinephrine and/or norepinephrine for at least 2 hours during the first 24 hours | 34 (23%) | 25 (38%) | 0.03 | - |
| Acute Kidney Insufficiency at H6****** | 58 (38%) | 43 (65%) | 0.0004 | - |
| SAPSII | 48.7 ± 16.0 | 63.5 ± 19.4 | <0.0001 | 1.04 (1,02-1.06)  (for 1 point-increase) |

*****: Patients placed on RRT between H9 and H72 or belonging to the « Failure » or « Injury » RIFLE classes were considered to have renal function impairment at H72. ****:** At H6 the patients were classified in two groups according to the *creatinine criterion* of the RIFLE classification taking into account the highest value of serum creatinine between H1 and H6. Patients of the “Risk”, “Injury” or “Failure” classes were considered as having AKI at H6.

AKI: acute kidney insufficiency; MAP: mean arterial pressure; NSAID: non steroidal anti inflammatory drug; RRT: renal replacement therapy; SAPS: simplified acute physiology score

**Table A2**: Search of potential interactions between AKI at H6, septic shock and chronic hypertension, linked to the occurrence of AKI at H72

| Variables entered as potential predictors of AKI at H72 | Adjusted Odd ratios for variables remaining as potential predictors after multivariate *stepwise* logistic regression |
| --- | --- |
| MAP averaged over H1 to H12 (mmHg) | Not retained |
| MAP averaged over H1 to H24 (mmHg) | Not retained |
| MAP averaged over H6 to H24 (mmHg) | Not retained |
| MAP averaged over H12 to H24 (mmHg) | **0.95 (0.92- 0.98)**  (for 1 mmHg) |
| Use of NSAIDs | Not retained |
| Highest serum creatinine between H1 and H6 (µmom/L) | **1.003 (1.0002-1.0067)**  (for 1 µmol/L) |
| Liver cirrhosis | **6.8 (1.4-33.7)**  (for 1 point-increase) |
| More than 0.5 µg/kg/min of epinephrine and/or norepinephrine for at least 2 hours the first 24 hours | Not retained |
| Acute Kidney Insufficiency at H6 | Not retained |
| SAPSII | **1.04 (1.02-1.06)**  (for 1 point-increase) |
| “At least 2 conditions among chronic hypertension, septic shock, presence of AKI at H6” | **2.43 (1.16 – 5.09)** |
| Chronic hypertension *and* septic shock | Not retained |
| Chronic hypertension *or* septic shock | Not retained |
| Chronic hypertension *and* AKI at H6 | Not retained |
| Chronic hypertension *or* AKI at H6 | Not retained |
| Septic shock *and* AKI at H6 | **0.34 (0.12 – 0.93)** |
| Septic shock *or* AKI at H6 | Not retained |
| Chronic hypertension *and* septic shock *and* AKI at H6 | Not retained |
| Chronic hypertension *or* septic shock *or* AKI at H6 | Not retained |
| Septic shock | Not retained |
| Chronic hypertension | Not retained |

AKI: acute kidney insufficiency; MAP: mean arterial pressure; NSAID: non steroidal anti inflammatory drug;

SAPS: simplified acute physiology score

**2) Evolution of MAP during the first 24 hours compared between patients with and without AKI at H72, in subgroups of patients according to the presence or not of chronic hypertension.**

We first examined the potential effect of chronic hypertension on MAP evolution and on the difference in MAP evolution between patients with and without acute kidney insufficiency (AKI) at H72 by 4-factor analysis of variance for repeated measurements, with MAP at each time point between H1 and H24 as the dependent variable. The 4 independent factors entered were AKI at H72, AKI at H6, septic shock and chronic hypertension.

In our whole population this analysis disclosed that only the factors “AKI at H72” (p<0.0001) and “AKI at H6” (p<0.0001) were significantly linked to the MAP evolution, whereas the factors “septic shock” (p=0.25) and “chronic hypertension” (p=0.34) seemed to have no influence on MAP evolution. However, all the possible combinations of the presence or not of the 4 factors entered in the analysis showed that they interacted to influence the MAP evolution during the first 24 hours (p<0.02 in all cases). This was due to different effects of chronic hypertension on the difference in MAP evolution between patients with and without AKI at H72, according to the presence or not of AKI at H6 or of septic shock. This is illustrated in **Figures A1**, **A2** and **A3** that display the compared evolution of MAP during the first 24 hours between patients who showed AKI at H72 and those who did not, in different sub-groups acording to the presence or not of chronic hypertension: First, in **Figure A1**, the difference in MAP between patients with or without AKI at H72 is less obvious in the patients with chronic hypertension, although significant at some time points. Second, no difference was observed in MAP evolution between patients with and without AKI at H72 in patients with chronic hypertension and either AKI at H6 or no AKI at H6 (**Figure A2**), while in patients with septic shock, although it did not reach staistical significance, patients with chronic hypertension who developed AKI at H72 tended to have lower MAP compared to patients with chronic hypertension and no AKI at H72 (**Figure A3**).

We did not push further the subgroup analysis, for instance by subdviding our study population in 8 subgroups according to the presence or not of AKI at H6, septic shock and chronic hypertension, because samples sizes would have been too small to allow interpretation of the results.

From our observations, with regard to the link between chronic hypertension and the potential influence of MAP on the occurrence of AKI at H72, we made the following interpretations:

Our main result (see “Results” section), i.e. that in shocked patients with initial renal insult the occurrence of AKI at H72 is linked to the time-averaged MAP during the 24 hours of care, is confirmed in patients *without* chronic hypertension, while our study was underpowered to conclude it was not also true in patients with chronic hypertension. Besides the too small size of our study population to draw firm conclusions, it must be noted that patients with chronic hypertension constitute a very inhomogeneous group in that sense that baseline stable arterial pressure is rarely known on admission and probably different between patients, and that they do not receive the same regular antihypertensive (combinations of) medications. Further, it is possible that our poulation did not comprise a sufficient number of chronic hypertensive patients in whom a high enough MAP (sufficient to avoid the worsening of renal function) could be attained with therapy. Therefore, we cannot exclude that chronic hypertensive patients with shock really need far higher MAP than the classical 65 mmHg, and even far higher than the 72-82 mmHg level we observed, in order to avoid AKI.

**Figures legends**

**Figure A1:**

**Title**: Evolution of mean arterial pressure (MAP) during the first 24 hours compared between patients with and without acute kidney insufficiency at H72, in patients with and without chronic hypertension

**Legend:**

The evolution of hourly MAP compared between patients who will have acute kidney insufficiency (AKI) at H72 (**black squares**) and those who will not (**open squares** ), is shown for the patients without (**left panel**) and with chronic hypertension (**right panel**).

Asterisks upon time points indicate a significant difference (p<0.05) between patients who will have AKI at H72 and those who will not (*post hoc* comparison after analysis of variance).

Error bars represent standard errors

**Figure A2:**

**Title**: Evolution of mean arterial pressure (MAP) during the first 24 hours in sub-groups of patients according to the presence or not of acute kidney insufficiency at H6 and to the presence or not of chronic hypertension

**Legend:**

The evolution of hourly MAP compared between patients who will have acute kidney insufficiency (AKI) at H72 (**black squares**) and those who will not (**open squares** ), is shown for the patients without (**left panels**) and with chronic hypertension (**right panels**), according to the presence (**upper panels**) or not (**lower panels**) of AKI at H6.

Asterisks upon time points indicate a significant difference (p<0.05) between patients who will have AKI at H72 and those who will not (*post hoc* comparison after analysis of variance).

Error bars represent standard errors

**Figure A3:**

**Title**: Evolution of mean arterial pressure (MAP) during the first 24 hours in sub-groups of patients according to the presence or not of septic shock and to the presence or not of chronic hypertension

**Legend:**

The evolution of hourly MAP compared between patients who will have acute kidney insufficiency (AKI) at H72 (**black squares**) and those who will not (**open squares** ), is shown for the patients without (**left panels**) and with chronic hypertension (**right panels**), according to the presence (**upper panels**) or not (**lower panels**) of septic shock.

Asterisks upon time points indicate a significant difference (p<0.05) between patients who will have AKI at H72 and those who will not (*post hoc* comparison after analysis of variance).

Error bars represent standard errors

**Figure A1**


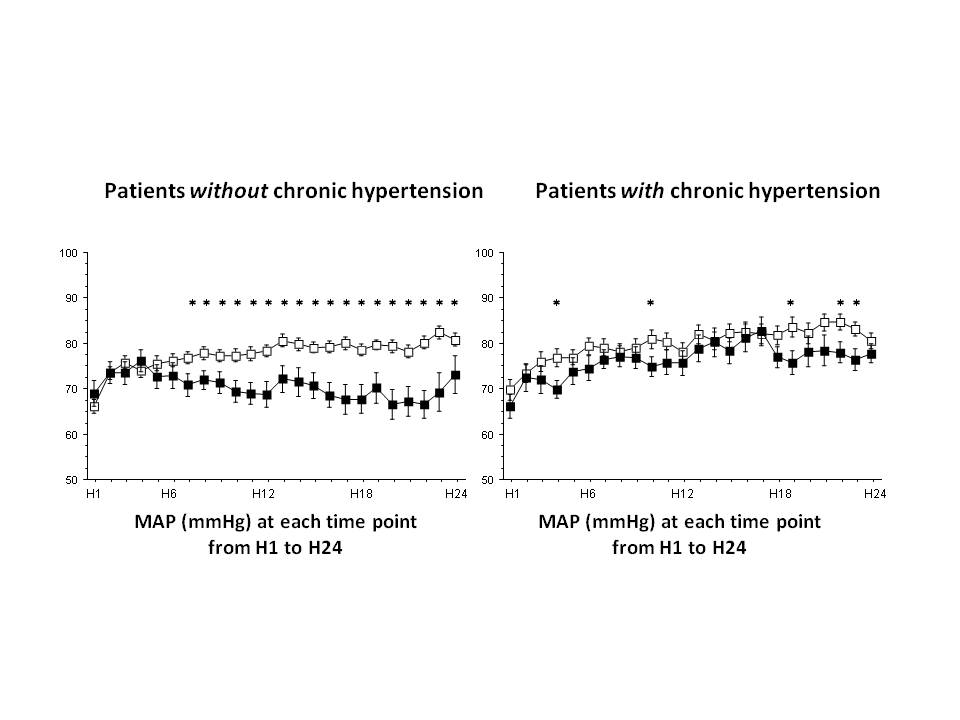


**Figure A2**


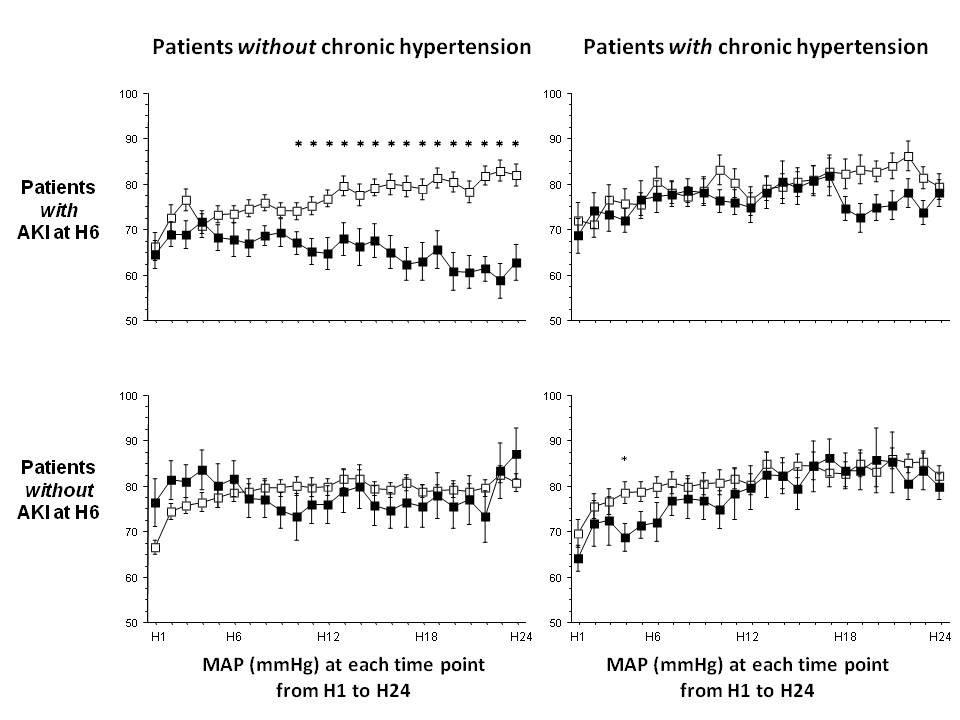


**Figure A3**


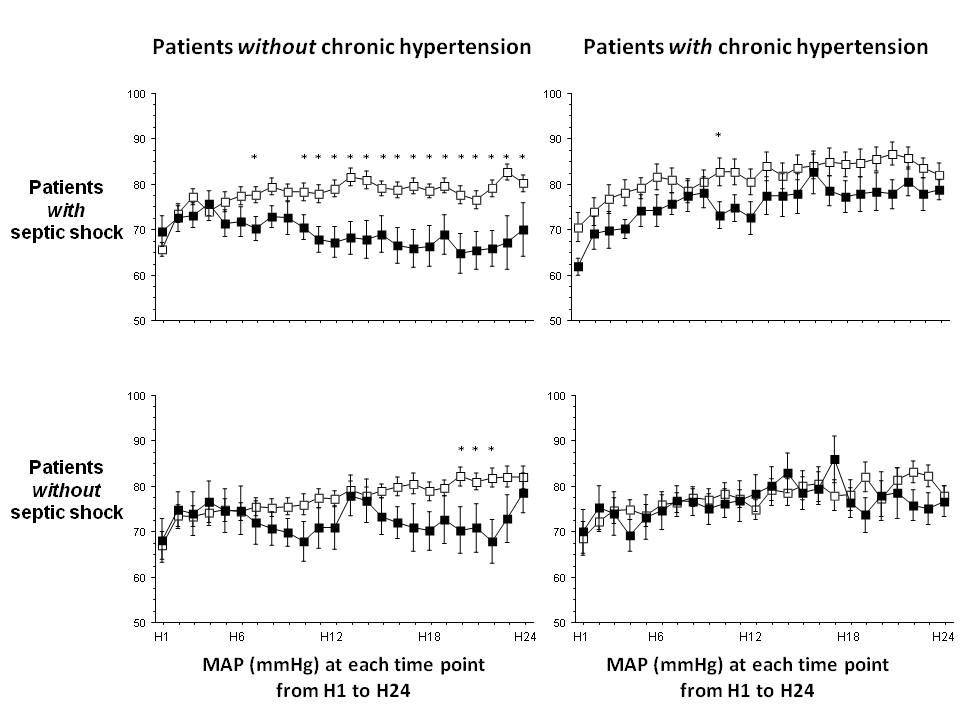

Supplement: Additional file 1 — The interrelationships between chronic arterial hypertension, mean arterial pressure during shock, and the occurrence of Acute Kidney Insufficiency. 1) Relation between chronic hypertension and Acute Kidney Insufficiency; 2) Evolution of mean arterial pressure during the first 24 hours compared between patients with and without Acute Kidney Insufficiency at H72, in subgroups of patients according to the presence or not of chronic hypertension. [file cc10253-S1.DOC]
